# *Arabidopsis WRKY2 *transcription factor mediates seed germination and postgermination arrest of development by abscisic acid

**DOI:** 10.1186/1471-2229-9-96

**Published:** 2009-07-22

**Authors:** Wenbo Jiang, Diqiu Yu

**Affiliations:** 1 Key Laboratory of Tropical Forest Ecology, Xishuangbanna Tropical Botanical Garden, Chinese Academy of Sciences, Kunming, Yunnan 650223, PR China; 2 Graduate University of Chinese Academy of Sciences, Beijing 100049, PR China

## Abstract

**Background:**

Plant WRKY DNA-binding transcription factors are key regulators in certain developmental programs. A number of studies have suggested that WRKY genes may mediate seed germination and postgermination growth. However, it is unclear whether WRKY genes mediate ABA-dependent seed germination and postgermination growth arrest.

**Results:**

To determine directly the role of *Arabidopsis WRKY2 *transcription factor during ABA-dependent seed germination and postgermination growth arrest, we isolated T-DNA insertion mutants. Two independent T-DNA insertion mutants for *WRKY2 *were hypersensitive to ABA responses only during seed germination and postgermination early growth. *wrky2 *mutants displayed delayed or decreased expression of *ABI5 *and *ABI3*, but increased or prolonged expression of *Em1 *and *Em6*. *wrky2 *mutants and wild type showed similar levels of expression for *miR159 *and its target genes *MYB33 *and *MYB101*. Analysis of *WRKY2 *expression level in ABA-insensitive and ABA-deficient mutants *abi5-1*, *abi3-1*, *aba2-3 *and *aba3-1 *further indicated that ABA-induced *WRKY2 *accumulation during germination and postgermination early growth requires *ABI5*, *ABI3*, *ABA2 *and *ABA3*.

**Conclusion:**

ABA hypersensitivity of the *wrky2 *mutants during seed germination and postgermination early seedling establishment is attributable to elevated mRNA levels of *ABI5*, *ABI3 *and *ABI5*-induced *Em1 *and *Em6 *in the mutants. *WRKY2*-mediated ABA responses are independent of *miR159 *and its target genes *MYB33 *and *MYB101*. *ABI5*, *ABI3*, *ABA2 *and *ABA3 *are important regulators of the transcripts of *WRKY2 *by ABA treatment. Our results suggest that *WRKY2 *transcription factor mediates seed germination and postgermination developmental arrest by ABA.

## Background

Abscisic acid (ABA) is a phytohormone regulating plant responses to a variety of environmental stress, particularly water deprivation, notably by regulating stomatal aperture [1-4]. It also plays an essential role in mediating the initiation and maintenance of seed dormancy [5]. Late in seed maturation, the embryo develops and enters a dormant state that is triggered by an increase in the ABA concentration. This leads to the cessation of cell division and activation of genes encoding seed storage proteins and proteins required to establish desiccation tolerance [6]. Exposure of seeds to ABA during germination leads to rapid but reversible arrest in development. ABA-mediated postgermination arrest allows germinating seedlings to survive early water stress [5]. Based on ABA inhibition of seed germination, mutants with altered ABA sensitivity have been identified. These screens have led to the identification of several ABA-insensitive genes [7-13]. The transcription factors *ABI3 *and *ABI5 *are known to be important regulators of ABA-dependent growth arrest during germination [14,15]. *ABI5*, an ABA-insensitive gene, encodes a basic leucine zipper transcription factor. Expression of *ABI5 *defines a narrow developmental checkpoint following germination, during which *Arabidopsis *plants sense the water status in the environment. *ABI5 *is a rate-limiting factor conferring ABA-mediated postgermination developmental growth arrest [5].*ABI3 *is also reactivated by ABA during a short development window. Like *ABI5*, *ABI3 *is also required for the ABA-dependent postgermination growth arrest [15]. However, *ABI3 *acts upstream of *ABI5 and *is essential for *ABI5 *gene expression [15]. In arrested, germinated embryos, ABA can activate de novo late embryogenesis programs to confer osmotic tolerance. During a short development window, *ABI3*, *ABI5 *and late embryogenesis genes are reactivated by ABA. ABA can activate *ABI5 *occupancy on the promoter of several late embryogenesis-abundant genes, including *Em1 *and *Em6 *and induce their expression [15,16]. On the other hand, ABA-induced *miR159 *accumulation requires *ABI3 *but is only partially dependent on *ABI5*. *MYB33 *and *MYB101*, two *miR159 *targets, are positive regulators of ABA responses during germination and are subject to ABA-dependent *miR159 *regulation [17].

The family of plant-specific WRKY transcription factors contains over 70 members in *Arabidopsis thaliana *[18-20]. WRKY proteins typically contain one or two domains composed of about 60 amino acids with the conserved amino acid sequence WRKYGQK, together with a novel zinc-finger motif. WRKY domain shows a high binding affinity to the TTGACC/T W-box sequence [21]. Based on the number of WRKY domains and the pattern of the zinc-finger motif, WRKY proteins can be divided into 3 different groups in *Arabidopsis *[20].

A growing body of studies has shown that WRKY genes are involved in regulating plant responses to biotic stresses. A majority of reported studies on WRKY genes address their involvement in disease responses and salicylic acid (SA)-mediated defense [20,22-25]. In addition, WRKY genes are involved in plant responses to wounding [26]. Although most WRKY proteins studied thus far have been implicated in regulating biotic stress responses, some WRKY genes regulate plant responses to freezing [27], oxidative stress [28], drought, salinity, cold, and heat [29-31].

There is also increasing evidence indicating that WRKY proteins are key regulators in certain developmental programs. Some WRKY genes regulate biosynthesis of anthocyanin [32], starch [33], and sesquiterpene [34]. Other WRKY genes may regulate embryogenesis [35], seed size [36], seed coat and trichome development [32,37], and senescence [38-40].

A number of studies have suggested that WRKY genes may mediate seed germination and postgermination growth. For example, wild oat WRKY proteins (ABF1 and ABF2) bind to the box2/W-box of the GA-regulated *α-Amy2 *promoter [41]. A barley WRKY gene, *HvWRKY38*, and its rice (*Oryza sativa*) ortholog, *OsWRKY71 *act as a transcriptional repressor of gibberellin-responsive genes in aleurone cells [42]. However, it is unclear whether WRKY genes mediate ABA-dependent seed germination and postgermination growth arrest. In this study, we report that *wrky2-1 *and *wrky2-2 *mutants are hypersensitive to ABA during germination and postgermination early growth. We have analyzed genetic interactions between the *wrky2 *mutant and the *abi3-1 *and *abi5-1 *mutants and found that ABA hypersensitivity of the *wrky2 *mutants is attributable to increased mRNA levels for *ABI5*, *ABI3 *and *ABI5*-induced *Em1 *and *Em6*. Furthermore, *ABI5*, *ABI3*, *ABA2 *and *ABA3 *are important regulators of ABA-induced expression of *WRKY2*.

## Results

### *WRKY2 *T-DNA insertion mutants are hypersensitive to ABA during seed germination and postgermination early growth

**Figure 1:**
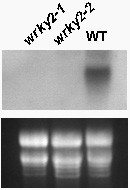
**Identification of *wrky2 *mutants by northern blot analysis**. RNA was extracted from the seedlings that have grown on MS medium with 1.5 μM ABA 4 days after the end of stratification. Each lane contained 20 μg total RNA. Each experiment also was executed three times.

To analyze the molecular events in ABA-regulated germination and seedling growth, we sought to identify WRKY transcription factors associated with these growth and developmental stages. For this purpose, we screened T-DNA insertion mutants for a number of *Arabidopsis WRKY *genes for altered sensitivity to ABA based on their germination rates and seedling growth in MS media containing ABA. As shown in Figure 1, *WRKY2 *T-DNA insertion mutants *wrky2-1 *(Salk_020399) and *wrky2-2 *(Sail_739_F05) accumulated no *WRKY2 *transcript of expected size and exhibited increased sensitivity to ABA during seed germination and seedling growth. The two mutants display no other obvious phenotypes in morphology, growth, development or seed size (data not shown).

**Figure 2:**
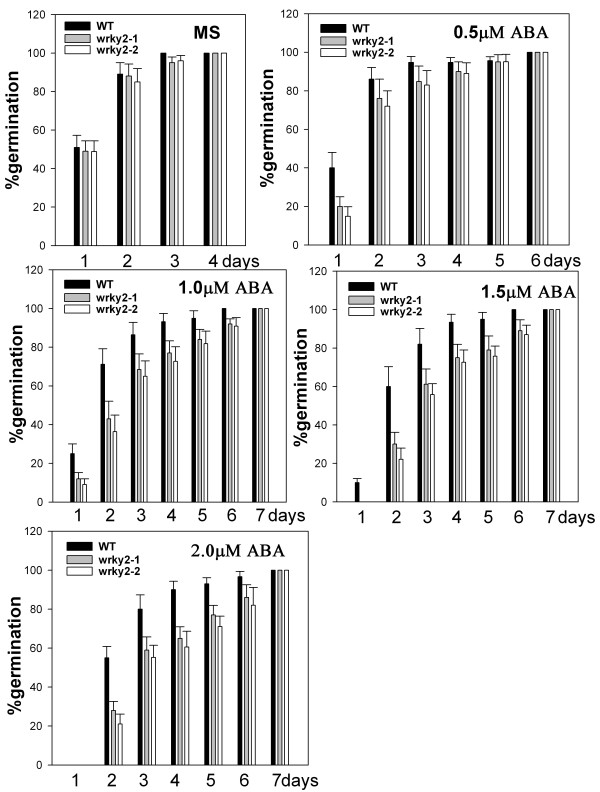
**ABA dose-response analysis of germination in *wrky2-1 and wrky2-2 *mutants**. Seeds were germinated on MS plates containing 0 μM, 0.5 μM, 1.0 μM, 1.5 μM, and 2.0 μM ABA. Plates were routinely kept for 3 days in the dark at 4°C and transferred to a tissue culture room under constant light at 22°C. Germination efficiencies (radicle emergence) of wild type and *wrky2 *mutants seeds for 7 d after stratification. Three independent experiments are shown, and above 100 seeds were used in each experiment.

**Figure 3:**
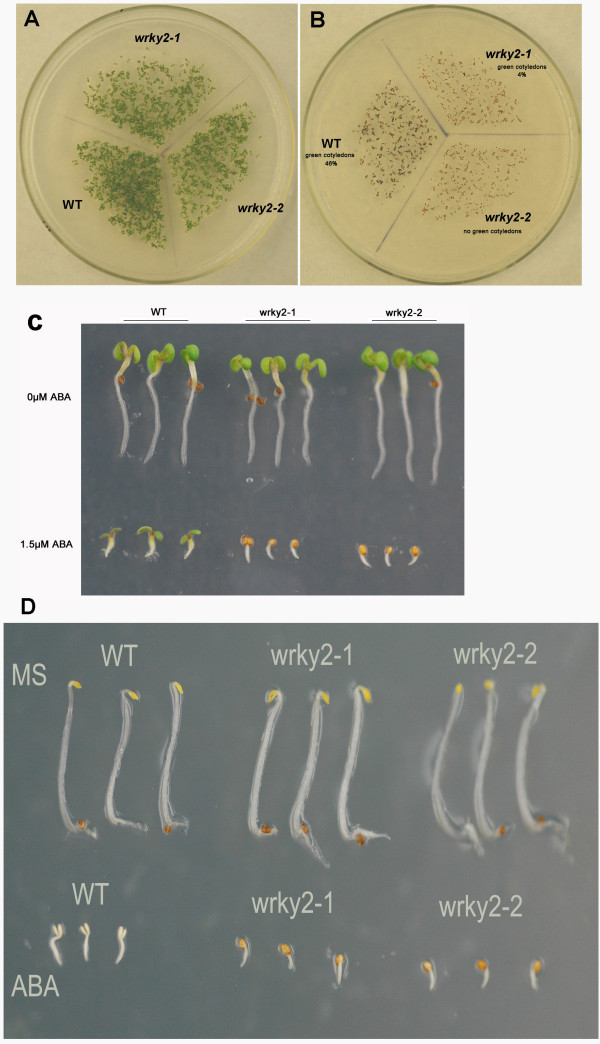
***wrky2 *mutants are hypersensitive to ABA responses during postgermination growth**. (A) Photographs of WT, *wrky2-1 *and *wrky2-2 *seedlings on MS medium at 7 d after the end of stratification. (B) Photographs of WT, *wrky2-1 *and *wrky2-2 *seedlings on MS medium with 1.5 μM ABA at 7 d after stratification. (C) Photographs of representative examples in A and B. (D) Photographs of representative examples in A and B in darkness.

**Figure 4:**
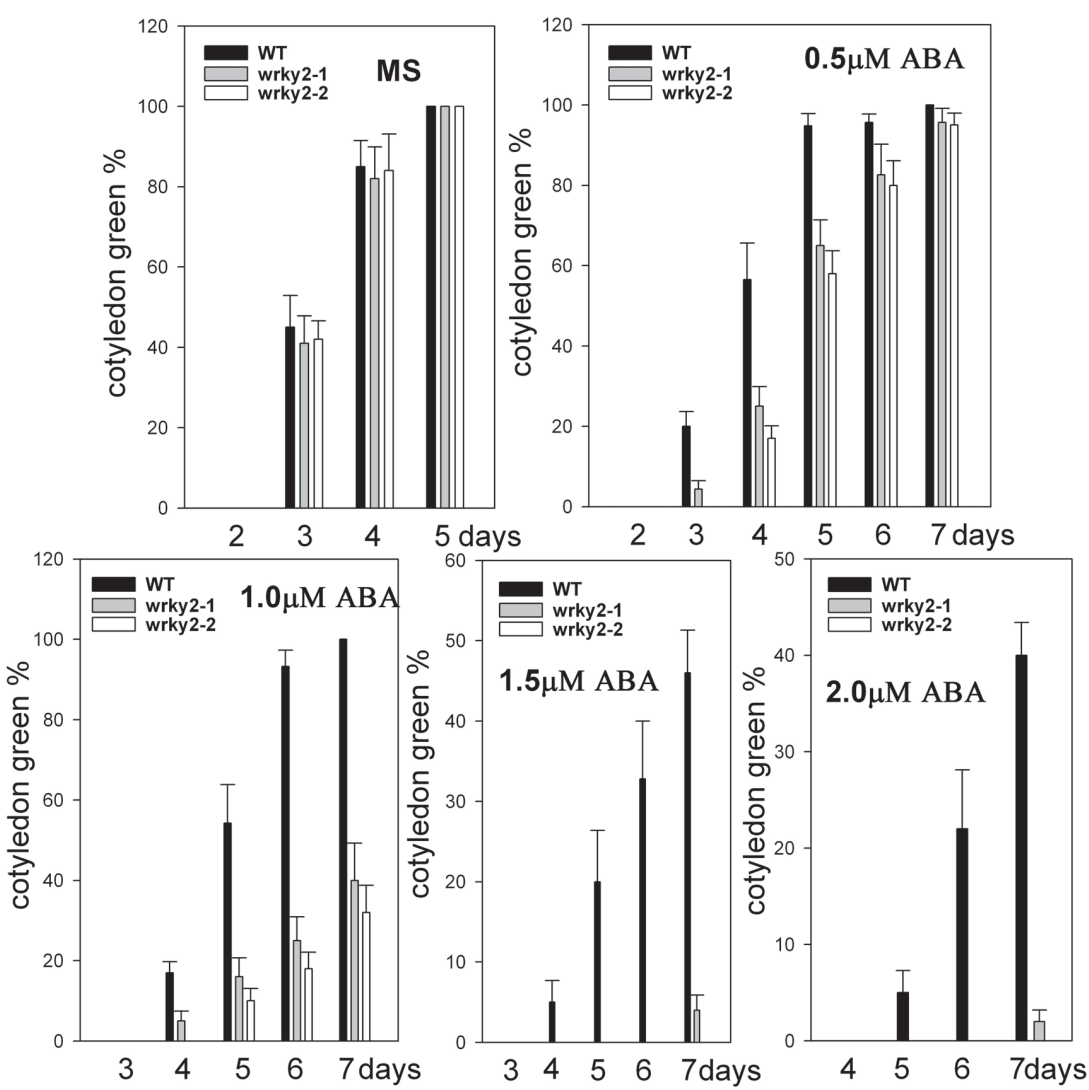
***wrky2 *mutants are hypersensitive to ABA responses during postgermination growth Seeds were germinated**. Postgermination growth efficiencies (green cotyledons) were scored for 7 d after stratification. Three independent experiments are shown, and above 100 seeds were used in each experiment.

To determine the role of *WRKY2 *in seed germination and early seedling growth, wild-type and *wrky2 *mutant seeds were germinated on MS medium containing 0 μM, 0.5 μM, 1.0 μM, 1.5 μM, 2.0 μM ABA, and compared for differences in germination and postgerminative growth. In the absence of ABA, there was no significant difference in germination between wild-type and mutant seeds (Figure 2 and Figure 3A). In the presence of ABA, both mutants germinated later than wild type. On MS medium with 0.5 μM and 1.0 μM, 40% and 25% of wild-type seeds germinated after one day, respectively. At these two concentrations, the germination rates of the two mutants were only about half of wild type (Figure 2). On MS medium with 1.5 μM, 10% of wild-type seeds still germinated, but no *wrky2-1 *and *wrky2-2 *seeds germinated. Likewise, significantly more wild-type seeds germinated than the mutant seeds after two days on MS medium with 1.5 μM and 2.0 μM ABA (Figure 2). Early seedling growth of both mutants was also slower than that of wild type. After 7 d, 46% of wild-type but only 4% of *wrky2-1 *and none of wrky2-2 mutants had green cotyledons on MS medium with 1.5 μM ABA (Figure 4, 3B and 3C). These results show that *wrky2 *mutants are hypersensitive to ABA responses during germination and postgermination growth.

To analyze the role of *WRKY2 *during the seedling growth stages, we first germinated the wild-type and *wrky2 *mutant seeds on MS medium for 4 d and then transferred the seedlings onto MS medium containing 0 μM, 0.5 μM, 1.0 μM, 1.5 μM, 2.0 μM, 5.0 μM, 10 μM, 20 μM, 40 μM and 80 μM ABA. No significant difference was observed between the wild type and *wrky2 *mutants 10 days after the transfer (data not shown).

When the germination experiments were carried out in dark, *wrky2 *mutants are again more sensitive to ABA responses than wild type (Figure 3). Taken together, these observations suggest that *wrky2 *mutants are hypersensitive to ABA responses only during seed germination and early seedling growth.

### Response of *wrky2 *mutants to ABA defines a limited developmental window

**Figure 5:**
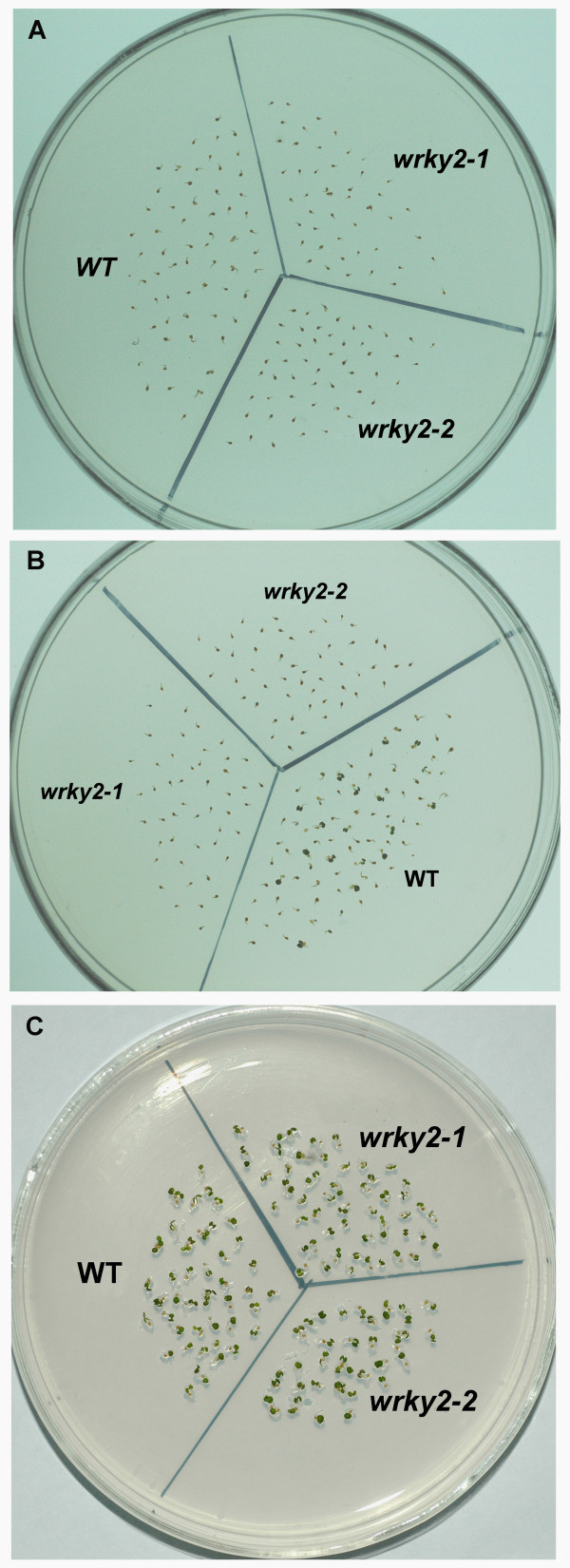
***wrky2 *mutants are hypersensitive to ABA responses in a short development window**. (A) Seeds were germinated on MS medium 1 d after stratification, all were transferred to MS medium with 5 μM ABA. Photographs were taken 6 d after transfer. (B) Seeds were germinated on MS medium and 2 d after stratification, all were transferred to MS medium with 5 μM ABA. Photographs were taken 5 d after transfer. (C) Seeds were germinated on MS medium and 3 d after stratification, all were transferred to MS medium with 5 μM ABA. Photographs were taken 4 d after transfer.

The transcription factors *ABI3 *and *ABI5 *are known to be important regulators of ABA-dependent growth arrest during germination [14,15]. *ABI3 *and *ABI5 *are reactivated by ABA during a short development window. Given the ABA hypersensitivity of *wrky2 *mutants, we studied the effect of ABA on the early development of *wrky2 *mutants. Application of 5 μM ABA [5] within 48 h post-stratification maintained the germinated embryos of *wrky2 *mutants in an arrested state for several days, but did not prevent germination, whereas a significant percentage of wild-type germinated embryos escaped growth arrest and turned green (Figure 5A and 5B). ABA applied outside the 48-h time frame failed to arrest growth and prevent greening (Figure 5C). If the seeds were transferred to ABA-containing media immediately or one day after stratification, we observed that the *wrky2 *mutants were more sensitive to ABA responses than the wild type. However, if the seeds were transferred to ABA-containing media 2 days post-stratification, there was no significant difference between the wild type and *wrky2 *mutants. These results indicated that *wrky2 *mutants were hypersensitive to ABA responses for a short development window.

### *WRKY2 *mediates signal pathway of ABA-dependent germination and postgermination early growth

**Figure 6:**
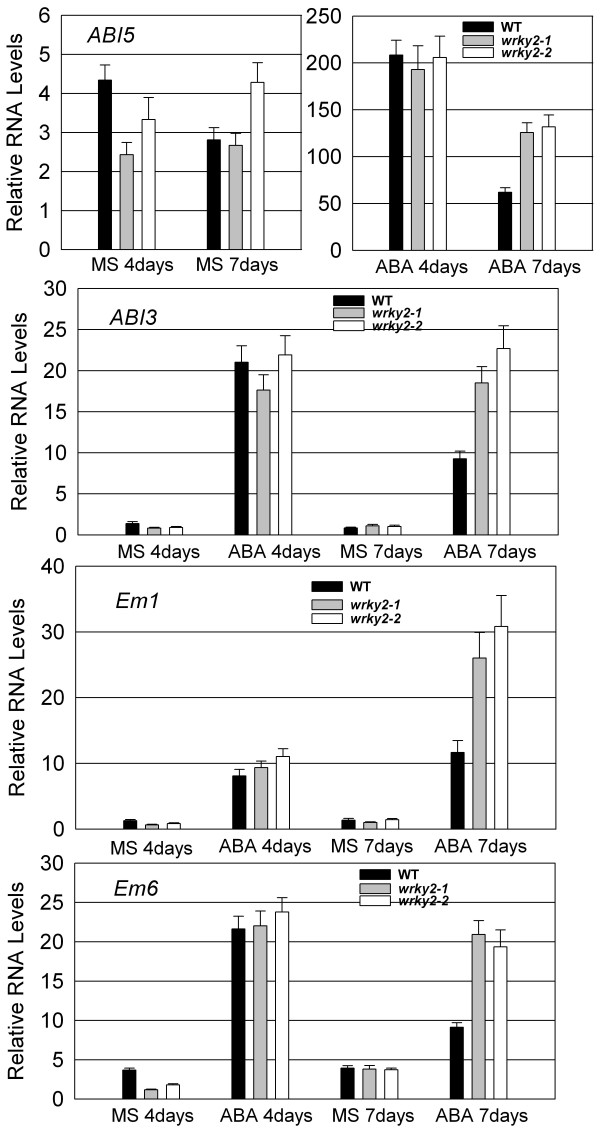
**RNA levels of *ABI5*, *ABI3*, *Em1 *and *Em6 *in *wrky2 *mutants and wild type**. RNA was extracted from seedlings on MS medium without ABA or with 1.5 μM ABA 4 d or 7 d after the end of stratification. Relative RNA levels of 4 genes were analyzed using gene-specific primers by real-time PCR. Three independent experiments are shown by reextracting RNA from other samples. Each experiment also was executed three times.

During germination, ABA can activate de novo late embryogenesis programs to confer osmotic tolerance in arrested, germinated embryos [15]. During a short development window, *ABI3*, *ABI5 *and late embryogenesis genes are reactivated by ABA. *ABI3 *acts upstream of *ABI5 *and is essential for *ABI5 *gene expression. ABA induces *ABI5 *occupancy on the promoter of *Em1 *and *Em6*, and activates these late embryogenesis-abundant genes [15,16]. To analyze the expression of these genes in *wrky2 *mutants, we germinated the wild type and *wrky2 *mutants on MS medium with 0 and 1.5 μM ABA for 4 or 7 days. Total RNA was isolated and analyzed using quantitative RT-PCR with gene-specific primers. As shown in Figure 6, when these seedlings have grown on MS medium without ABA 4 days post-stratification, expression of *ABI5*, *ABI3*, *Em1 *and *Em6 *was reduced in the *wrky2 *mutants relative to that in the wild type. At 7 days post-stratification, wild type and *wrky2 *mutants accumulated similar levels of transcripts for *ABI3*, *ABI5*, *Em1 *and *Em6 *(Figure 6).

On the other hand, on medium containing ABA, the expression levels of *ABI5*, *ABI3*, *Em1 *and *Em6 *were similar in the wild type and *wrky2 *mutants at 4 days post-stratification. However, at 7 days post-stratification, there were higher expression levels of these genes in *wrky2 *mutants than in the wild type, which was consistent with microarray analysis (Figure 6 and Table 1). By contrast at 4 days post-stratification, the expression of *ABI3 *and *Em6 *didn't decrease or decreased only slightly in *wrky2 *mutants, but decreased rapidly in the wild type within 7 days post-stratification. Within 7 days post-stratification, expression of *AIB5 *decreased faster in wild type than in *wrky2 *mutants. Expression of *Em1 *increased in *wrky2 *mutants 7 days post-stratification, but was unchanged in wild type (Figure 6).

**Table 1:** Microarray analysis of *wrky2 *mutants and wild type

Microarray data of 6 genes		
Gene	Transcript ID	The ratio of *wrky2 *mutants vs WT

*ABI5*	At2G36270	2.30
*ABI3*	At3G24650	6.06
*Em1*	At3G51810	4.29
*Em6*	At2G40170	5.28
*MYB33*	At5G06100	1.15
*MYB101*	At2G32460	0.62

RNA was extracted from seedlings of *wrky2 *mutants and wild type on MS medium with 1.5 μM ABA 7 d post-stratification. Relative RNA levels of *ABI5*, *ABI3*, *Em1*, *Em6*, *MYB33 *and *MYB101 *were analyzed by Microarray data.

These results indicated that *wrky2 *mutants displayed delayed or decreased expression of *ABI5 *and *ABI3*, but increased or prolonged expression of *Em1 *and *Em6*.

### The expression of *WRKY2 *in ABA-insensitive mutants and ABA-deficient mutants

**Figure 7:**
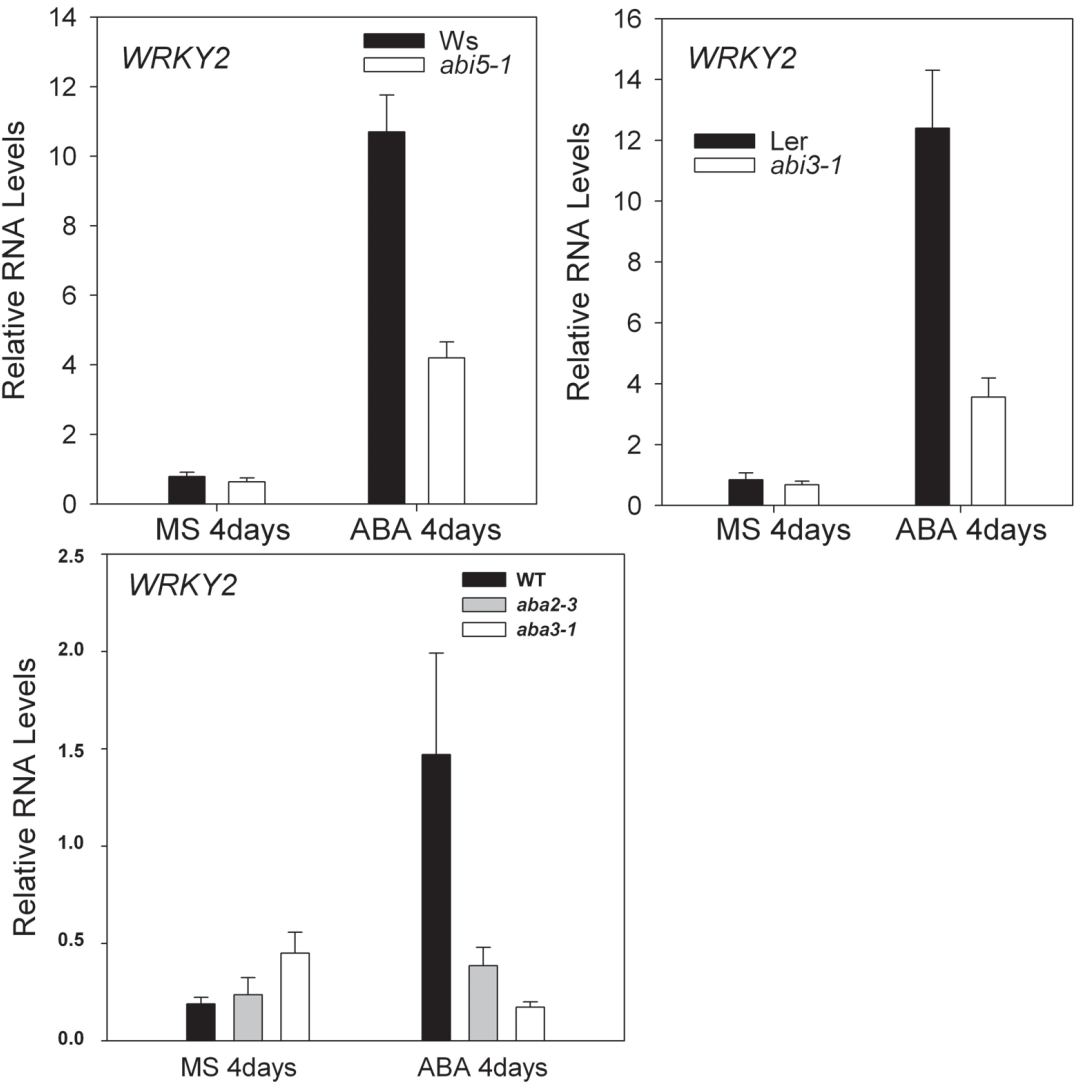
**RNA levels of WRKY2 in abi5-1, abi3-1, aba2-3 and aba3-1 mutants**. RNA was extracted from seedlings on MS medium without ABA or with 1.5 μM ABA 4 d post-stratification. Relative RNA levels of *WRKY2 *were analyzed using gene-specific primers by real-time PCR. Three independent experiments are shown by reextracting RNA from other samples. Each experiment also was executed three times.

Because *wrky2 *mutants are more sensitive to ABA during seed germination and postgermination growth arrest than the wild type, and *wrky2 *mutants also affect important genes of the ABA signal pathway in the regulation of germination and postgermination growth, we analyze whether *abi3-1*, *abi5-1*, *aba2-3 *and *aba3-1 *mutants have an effect on the expression of *WRKY2*. We germinated all seeds on MS medium with 0 or 1.5 μM ABA for 4 days post-stratification. We performed quantitative RT-PCR with gene-specific primers. In the absence of ABA, the expression level of *WRKY2 *in the wild type (Ws) was 1.2 times of that in the *abi5-1 *mutants. In the presence of ABA, the level of *WRKY2 *was 2.5-fold. On the other hand, ABA treatment led to 13.6-fold increase in accumulation of *WRKY2 *in the wild type and 6.5-fold increase in *abi5-1 *mutants (Figure 7). These results suggest *ABI5 *is an important regulator of ABA-induced *WRKY2 *expression.

Without ABA, the level of *WRKY2 *in the wild type (Ler) was 1.2-fold higher than that in *abi3-1 *mutant. In the presence of ABA, the *WRKY2 *transcript level in wild type was 3.5-fold higher than that in *abi3-1 *mutant (Figure 7). These results indicate *ABI3 *maybe is a positive regulator of ABA-induced *WRKY2*. We also examined the expression level of *WRKY2 *in ABA-deficient *aba2-3 *and *aba3-1 *mutants [43,44]. As shown in Figure 7, with ABA, the expression level of *WRKY2 *in the wild type was 7.5 time higher than that without ABA, but the expression levels of *WRKY2 *in *aba2-3 *and *aba3-1 *mutants were only 1.6 and 0.38 times of those without ABA, respectively. On the other hands, without ABA, the expression levels of *WRKY2 *in *aba2-3 *and *aba3-1 *mutants were 1.2 and 2.3 times of that in the wild type. These observations show elevated expression of *WRKY2 *by ABA treatment requires *ABA2 *and *ABA3*. These results strongly suggest that *ABI5*, *ABI3*, *ABA2 *and *ABA3 *are important regulators of ABA-induced *WRKY2 *expression.

### The response of *WRKY2 *to ABA is independent of *miR159*, *MYB33 *and *MYB101*

**Figure 8:**
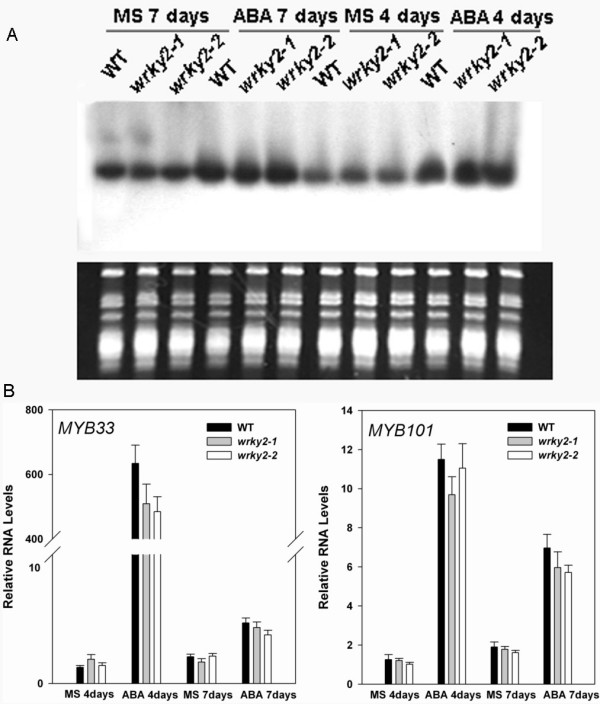
**RNA levels of *miR159*, *MYB33 *and *MYB101 *in *wrky2 *mutants and wild type**. RNA was extracted from seedlings on MS medium without ABA or with 1.5 μM ABA 4 d or 7 d post-stratification. (A) Each lane contained 20 μg total RNA. Each experiment also was executed three times. (B) Relative RNA levels of *WRKY2 *were analyzed using gene-specific primers by real-time PCR. Three independent experiments are shown by reextracting RNA from other samples. Each experiment also was executed three times.

It is known that ABA-induced *miR159 *accumulation requires *ABI3 *but is only partially dependent on *ABI5*. Furthermore, *MYB33 *and *MYB101*, which are *miR159 *targets, are positive regulators of ABA responses during germination and are subject to ABA-dependent *miR159 *regulation [17]. Figure 8A indicates that there was no significant difference in the level of mature *miR159 *between *wrky2 *mutants and the wild type. As shown in Figure 8B and Table 1, there was no difference in levels of transcripts of *MYB33 *and *MYB101 *either. These results indicate that *WRKY2*-mediated ABA signaling pathway is independent of *miR159 *and its target genes (*MYB33 *and *MYB101*) during seed germination and postgermination growth arrest.

## Discussion

The ability of exogenous ABA to arrest postgermination development has been used extensively to identify genes involved in ABA signaling [45]. In the present study, we discovered that *wrky2-1 *and *wrky2-2 *mutants were more sensitive than wild type to ABA responses during seed germination and postgermination early growth.

The *wrky2 *mutants are hypersensitive to ABA responses only within a short development window during seed germination and early seedling growth. During seed germination and early growth, the transcription factors *ABI3 *and *ABI5 *are known to be important regulators of ABA-dependent growth arrest, and their expression defines a narrow developmental checkpoint following germination [14,15]. *ABI5 *is a key player in ABA-triggered postgermination growth arrest, and the *abi5 *mutant seeds are insensitive to growth arrest by ABA, whereas seeds of *ABI5*-overexpressing lines are hypersensitive to ABA [5]. *ABI3 *acts upstream of *ABI5 *and is an important regulator of germination and postgermination growth by ABA [15]. ABA-induced *ABI5 *also occupies the promoter of *Em1 *and *Em6 *and activates these two late embryogenesis-abundant genes [15,16]. Lopez-Molina *et al*. (2001) have shown that application of 5 μM ABA within 60 h post-stratification in the wild type [5] (Ws) does not prevent germination, but arrest the germinated embryos for several days, during which both *ABI5 *transcripts and ABI5 proteins are detected at high levels. When applied outside the 60-h time frame, however, ABA fails to arrest growth and prevent greening and *ABI5 *transcripts and proteins are present at low levels [5]. Therefore, it is possible that the ABA-induced growth-arrest of *wrky2 *mutants might be associated with the expression level of *ABI5*. To test this hypothesis, we compared wild type and *wrky2 *mutants for analyzed ABA-regulated expression levels of *ABI5 *and *ABI5*-related important regulators during seed germination and postgermination development. We found that *wrky2 *mutants had higher mRNA levels of *ABI5*, *ABI3 *and *ABI5*-induced *Em1 *and *Em6 *than the wild type (Figure 6). The higher expression levels of these genes in the *wrky2 *mutants may be partly responsible for the enhanced growth arrest relative to that in the wild type in the presence of ABA (Figure 3 and 4).

With ABA treatment, *miR159 *accumulation requires *ABI3 *but is only partially dependent on *ABI5 *[17]. *MYB33 *and *MYB101*, which are *miR159 *targets, are positive regulators of ABA responses during germination [17]. Therefore we examined whether *WRKY2 *affected the mRNA levels of these genes. We found that the expression levels of these genes were not significantly different between *wrky2 *mutants and the wild type (Figure 8). These observations indicate that the response of *WRKY2 *to ABA during germination and early growth is independent of *miR159 *and its target genes (*MYB33 *and *MYB101*).

We also show that *ABI5*, *ABI3*, *ABA2 *and *ABA3 *are important positive regulators of ABA-induced *WRKY2 *accumulation (Figure 7). The expression levels of *WRKY2 *were elevated more drastically in the wild type than in *abi5-1 *and *abi3-1 *mutants after ABA treatment (Figure 7). This result indicates that these two genes are positive regulators of ABA-induced *WRKY2 *accumulation.

Genes encoding the enzymes for most of the steps of the ABA biosynthesis pathway have been cloned and their functions confirmed using ABA-deficient mutants for *ABA1 *[46], *ABA2 *[47,48], *ABA3 *[44,49,50] and *ABA4 *[51,52]. We analyzed the effect of ABA biosynthesis genes on *WRKY2 *transcripts using ABA-deficient *aba2-3 *and *aba3-1 *mutants [43,44]. We shows that elevated expression of *WRKY2 *after ABA treatment requires *ABA2 *and *ABA3*, indicating that these two genes are positive regulators of ABA-induced *WRKY2 *accumulation (Figure 7).

Several studies have shown that ABI5 binds to the ABA-responsive DNA elements (ABREs) with an ACGT core in the promoter of *Em1 *and *Em6*, and activates their expression [15,16]. On the other hand, other reports have shown that wild oat WRKY proteins (ABF1 and ABF2) bind to the box2/W-box of the GA-regulated *α-Amy2 *promoter [41], and *GaWRKY1 *highly activated the *CAD1-A *promoter by binding to W-box [34], and a barley WRKY gene, *HvWRKY38*, and its rice (*Oryza sativa*) ortholog, *OsWRKY71 *act as a transcriptional repressor of gibberellin-responsive genes in aleurone cells [42]. The promoter zone of *WRKY2 *has an ABA-responsive DNA element (CACGTGGC) containing an ACGT core, and the promoter zone of *ABI5 *has 6 W-box, whereas the promoter of *ABI3 *has 2 W-box. This raises the possibility that *WRKY2*, *ABI3 *and *ABI5 *are mutually regulated.

## Conclusion

Transcription factors *ABI5 *is an important regulator of postgermination developmental arrest mediated by ABA. Postgermination proteolytic degradation of the essential *ABI5 *transcription factor is interrupted by perception of an increase in ABA concentration, leading to *ABI5 *accumulation and reactivation of embryonic genes. Here we report that *wrky2-1 *and *wrky2-2 *mutants are more sensitive to ABA responses than the wild type during seed germination and postgermination early seedling establishment. ABA hypersensitivity of the *wrky2 *mutants is attributable to elevated mRNA levels of *ABI5*, *ABI3 *and *ABI5*-induced *Em1 *and *Em6 *in the mutants. *WRKY2*-mediated ABA responses are independent of *miR159 *and its target genes *MYB33 *and *MYB101*. ABA-induced *WRKY2 *accumulation during germination and postgermination early growth requires *ABI5*, *ABI3*, *ABA2 *and *ABA3*, suggesting that they are important regulators of the transcripts of *WRKY2 *by ABA treatment. Our results suggest that *WRKY2 *transcription factor mediates seed germination and postgermination developmental arrest by ABA.

## Methods

### Plant material and growth conditions

The *Arabidopsis thaliana *ecotypes Columbia, Wassilewskija and Landsberg erecta were used throughout this study. Seeds of the different genotypes of *Arabidopsis thaliana *were harvested from plants of the same age and stored for 5 weeks in the dark at 4°C. Seeds were surface-sterilized with 10% bleach and washed three times with sterile water. Sterile seeds were suspended in 0.1% agarose and plated on MS medium plus 1% sucrose. ABA (mixed isomers, Sigma) was added to the medium where indicated. Plates were routinely kept for 3 days in the dark at 4°C to break dormancy (stratification) and transferred thereafter to a tissue culture room under constant light at 22°C. Seeds of *abi5-1*, *abi3-1*, *aba2-3 *and *aba3-1 *were obtained from the Arabidopsis Biological Resource Center (ABRC) (Alonso et al., 2003).

### Identification of the *wrky2 *T-DNA Insertion Mutants

The *wrky2-1 *mutant (Salk_020399), obtained from the Arabidopsis Biological Resource Center (ABRC) (Alonso et al., 2003), contains a T-DNA insertion in the promoter of the *WRKY2 *gene, while *wrky2-2 *mutant (Sail_739_F05) is a gift of Dr. Zhixiang Chen (Department of Botany and Plant Pathology, Purdue University, West Lafayette, Indiana, USA). Homozygous plants of the *wrky2-1 *mutant were identified by two PCRs. In the first PCR, a pair of gene-specific primers designed to anneal outside of the T-DNA insertion were used, which in case of homozygosity does not produce a band of the predicted size (negative selection): forward primer 5'-ATCGTCATCATCTTCACCATTT-3' and reverse primer 5'-AACTGAAATCCTCAGTTCCGT-3'. In the subsequent PCR, the T-DNA border primer (5'-AAACGTCCGCAATGTGTTAT-3') in combination with forward primer in the first PCR. To confirm the nature and location of the T-DNA insertion, the PCR products were sequenced. To remove additional T-DNA loci or mutations from the mutants, backcrosses to wild-type plants were performed and plants homozygous for the T-DNA insertion were again identified.

### Quantitative Real-Time PCR

We germinated seeds of the wild type and *wrky2 *mutants on MS medium with or without 1.5 μM ABA for 4 or 7 days, and germinated the seeds of *abi5-1*, *abi3-1*, *aba2-3*, *aba3-1 *mutants and the wild type (Col, Ws and Ler) on MS medium with or without 1.5 μM ABA for 4 days. Harvest samples were froze immediately in liquid nitrogen, and stored at 80°C. RNA was extracted from these samples using An RNeasy Plant Mini kit (QIAgen, Valencia, CA), and DNA was removed via an on-column DNase treatment. For real-time PCR, the First Strand cDNA Synthesis kit (Roche, Diagnostics, Mannheim, Germany) was used to make cDNA from 1 μg of RNA in a 20 μL reaction volume. Each cDNA sample was diluted 1:20 in water, and 2 μL of this dilution was used as template for qPCR. Half-reactions (10 μL each) were performed with the Lightcycler FastStart DNA Master SYBR Green I kit (Roche, Mannheim, Germany) on a Roche LightCycler real-time PCR machine, according to the manufacturer's instructions. *ACT2 *(AT3G18780) was used as a control in qPCR. Gene-specific primers for detecting transcripts of *ACT2*, *WRKY2*, *ABI5*, *ABI3*, *Em1 *and *Em6 *are listed in Table 2. Gene-specific primers of *MYB33 *and *MYB101 *are as described by Allen *et al*. (2007) [53]. The qPCR reactions (10 μL each) for these genes contained the following: 1 μL SYBR Green I reaction mix, 3 mM MgCl_2_, 0.5 μM forward and reverse primers and 2 μL cDNA. The annealing temperature was 52°C in all cases. A no-template control was routinely included to confirm the absence of DNA or RNA contamination. The mean value of four replicates was normalized using the *ACT2 *gene as the control. Standard curves were generated using linearized plasmid DNA for each gene of interest. A second set of experiments was conducted on an independent set of tissue as a control.

**Table 2:** List of quantitative RT-PCR primer sequences

Quantitative RT-PCR primers		
Gene	Primer sequences (5'->3')	

*ABI5*	Primer forward	AGATGACACTTGAGGATTTCTTGGT
AT2G36270	Primer reverse	TGGTTCGGGTTTGGATTAGG
*ABI3*	Primer forward	CTGATTCTTGAATGGGTC
AT3G24650	Primer reverse	TTGTTATTAGGGTTAGGGT
*Em1*	Primer forward	CGGAGGAAGAAGGGATTGAGA
AT3G51810	Primer reverse	TGCCAAACACGGAACCTACA
*Em6*	Primer forward	GCAAAGAAGGGCGAGACC
AT2G40170	Primer reverse	TCCTCCTCAGCGTGTTCC
*WRKY2*	Primer forward	TTTCTTTGGGTTACGATG
AT5G56270	Primer reverse	CACAACAACTCTTGGCTC
*ACT2*	Primer forward	TGTGCCAATCTACGAGGGTTT
AT3G18780	Primer reverse	TTTCCCGCTCTGCTGTTGT

Quantitative RT-PCR primer sequences of *ABI5*, *ABI3*, *Em1*, *Em6*, *WRKY2 *and *ACT2*.

### Northern blotting

Total RNA was isolated using the TRIZOL reagent (BRL Life Technologies, Rockville, MD). 20 μg RNA was separated by electrophoresis on denaturing 17% polyacrylamide gels, and electroblotted onto a Hybond-N^+ ^membrane. The membrane was UV cross-linked and hybridized with PerfectHyb plus hybridization buffer (Sigma). DNA oligonucleotides complementary to *miR159 *were end-labeled using T4 polynucleotide kinase (Roche Applied Science, Penzberg, Germany). For RNA gel blot analysis of *WRKY2*, 20 μg total RNA was separated on 1.5% agarose-formaldehyde gels and blotted to nylon membranes. Blots were hybridized with [α-^32^P]dATP labeled gene-specific probes. Hybridization was performed in PerfectHyb plus hybridization buffer (Sigma) overnight at 68°C. The membrane was then washed for 10 minutes twice with 2× SSC (1× SSC is 0.15 M NaCl and 0.015 M sodium citrate) and 1% SDS and for 10 minutes with 0.1× SSC and 1% SDS at 68°C. Transcripts for *WRKY2 *were detected with about 1 kb before stop codon of *WRKY2 *cDNA as probe.

## Authors' contributions

WJ carried out all experiments of *WRKY2 *gene, participated in the design of the study, drafted and edit the manuscript. DY conceived of the study, participated in the design and helped to draft and edit the manuscript. All authors read and approved the final manuscript.
